# Three Shaft Industrial Gas Turbine Transient Performance Analysis

**DOI:** 10.3390/s23041767

**Published:** 2023-02-04

**Authors:** Waleligne Molla Salilew, Zainal Ambri Abdul Karim, Tamiru Alemu Lemma, Amare Desalegn Fentaye, Konstantinos G. Kyprianidis

**Affiliations:** 1 Mechanical Engineering Department, Universiti Teknologi PETRONAS, Seri Iskandar 32610, Malaysia; 2 School of Business, Society and Engineering, Mälardalen University, SE-72123 Västerås, Sweden

**Keywords:** gas turbine, design point, off-design, performance, transient, fouling, erosion

## Abstract

The power demand from gas turbines in electrical grids is becoming more dynamic due to the rising demand for power generation from renewable energy sources. Therefore, including the transient data in the fault diagnostic process is important when the steady-state data are limited and if some component faults are more observable in the transient condition than in the steady-state condition. This study analyses the transient behaviour of a three-shaft industrial gas turbine engine in clean and degraded conditions with consideration of the secondary air system and variable inlet guide vane effects. Different gas path faults are simulated to demonstrate how magnified the transient measurement deviations are compared with the steady-state measurement deviations. The results show that some of the key measurement deviations are considerably higher in the transient mode than in the steady state. This confirms the importance of considering transient measurements for early fault detection and more accurate diagnostic solutions.

## 1. Introduction

The gas turbine is a complex system that requires extensive effort to examine its performance and behaviour under different operating settings. It has been widely used in power generation and propulsion [1]. Predicting gas turbine performance and the behaviour of its components is crucial to taking the appropriate maintenance action. It will also be profoundly helpful in the design of new engines as well as for redesigning an existing product [2,3,4,5]. Gas turbine engines operate under harsh operating conditions that include high temperatures, pressures, and mechanical and thermal stresses; as a result, the performance of the gas-path components gradually deteriorates [6]. The compressor(s) and turbine(s), which are the main gas-path components, are the principal sources of performance issues for gas turbines because of their exposure to various internal and external degrading factors. Some of the most significant and likely current issues include a reduction in compressor efficiency caused by fouling, erosion, and object damage; a reduction in turbine efficiency caused by blade creep and erosion; a reduction in air flow capacity caused by fouling; and an increase in flow capacity caused by turbine erosion. The most common method for assessing engine performance is gas-path analysis (GPA), which detects potential faults and gives an early warning before they become more severe problems. An effective and reliable gas-path diagnostic tool that could detect, isolate, and assess potential problems based on the measurement deviations and suggest solutions well before they developed into more complex problems is therefore very essential. This plays a major role in the investment by ensuring high levels of gas turbine reliability and availability along with its best operating performance. There have been a variety of gas path diagnostic methods introduced so far, beginning with the traditional model-based (MB) methods (such as Kalman filter (KF) and gas path analysis (GPA)) to the most advanced artificial intelligence (AI)-based ones (such as the artificial neural network (ANN), expert system (ES), fuzzy logic (FL), Bayesian belief network (BBN), Deep Learning (DL), and Genetic Algorithm (GA)). In recent years, attention has been paid to hybrid methods.

Gas turbine performance prediction models primarily rely on gas path measurements obtained under steady-state operating conditions, but it is crucial to use a transient measurement for the real-time assessment of compressor surge margin [7]. Transient performance simulations are more sophisticated compared with steady-state performance models due to a large number of additional parameters, including inter-component volumes, rotor inertia, engine control, heat soakage, and tip clearance fluctuations [8]. Due to start-ups, load changes, shutdowns, and other environmental disturbances, gas turbines operate in transient mode. During these periods, most of the operating points fall below the design point, which will cause degradation or even compressor surge and stall [9]. When degradation occurs, component performance parameters (mass flow rate, pressure ratio, and isentropic efficiency) start changing from the nominal value, which can be represented by a shift in the characteristic curves on the component characteristic maps. Gas-path analysis (GPA) [10,11] is an inverse mathematical model that is used to estimate performance parameters’ deviation from gas-path measurements [12]. There are different approaches to developing a gas turbine simulation model. They are classified according to their complexity and accuracy [13]. Among these, component matching is the most reliable and accurate method that has gained acceptance and is utilised in most research works; for example, the work of Saravanamutto et al. [14], Muir et al. [15], Walsh et al. [16], and Kurzke [17].

Real-time fault diagnosis is growing into a key topic of research in gas turbine engine condition monitoring due to the significant impact of an unexpected failure. In this regard, transient models are essential complements to steady-state models in order to have more effective and accurate diagnostic solutions. Based on actual geometry and control logic, Poorya et al. [2] created a transient model to simulate the start-up operation of an industrial twin-shaft gas turbine. Using CFD simulation techniques and according to each component’s real geometry, the characteristic curves of the gas turbine’s components were produced. The developed engine performance model was linked with a simplified control logic and then tuned with field data. The outcomes demonstrate good agreement between the model outputs and the field data. SooYong et al. [18] developed a mathematical model for a multi-shaft gas turbine with an intercooler and regenerator to compute the static and dynamic characteristics of the engine. Chen et al. [7] presented a method for diagnosing gas turbines based on time-series data that includes both steady-state and transient operating conditions. By using time-series data that represent the observed deviations in the shaft rotational speed and calculating the power balance in the shaft, the surplus or deficit of power between the compressor and the turbine was quantified. During the dynamic maneuver, the maximum diagnostic errors reported for constant faults and sudden faults were less than 0.006%. Z. Li et al. [8] developed a performance model to support more accurate transient performance simulations of gas turbine engines at the conceptual and preliminary design stages. A novel transient performance simulation approach has been developed with generically simplified heat soakage and tip clearance models for major gas path components of gas turbine engines, including compressors, turbines, and combustors. The results were promising, as shown by comparisons between transient performance simulated with and without the heat soakage and tip clearing effects. The transient performance simulations and analysis performed during the conceptual and preliminary engine design stages are able to include the major heat soakage and tip clearance effects.

For predicting the transient performance of gas turbines, Djordje et al. [19] developed a real-time model. By comparing the simulation results with the experimental parameter results of the actual engine that had been empirically recorded, the developed dynamic model was confirmed. This includes the load changes and the start-up sequence. The system’s reaction to a step increase in fuel flow is covered by additional simulation. The simulation runs more quickly than the actual process does. Merrington [20] discussed the extraction of data for the diagnosis of gas turbines from transient data records and presented a novel method for obtaining fault diagnostic data from transient data recordings of gas turbine engines. Mehrpanahi et al. [21] used the dynamic model that was established to calculate the shaft’s RPMs throughout the load change and start-up phases. For the purpose of developing a fuel control strategy for the gas turbine starter, Ma et al. [22] have created a transient model. A dynamic model for a fighter jet engine was created by Khalid et al. [23], and the model contributed to control system optimization. Kim et al. [24] developed models for heavy-duty gas turbine transient analysis and presented dynamic simulation results of a modern gas turbine for the production of electric power. The operational behaviour of a whole engine is governed by the characteristics of the compressor, including the impact of variable vane technology, which has gained special attention. An implicit method is used to solve the sets of derivational equations numerically. To increase the accuracy and range of two-shaft gas turbine diagnostics under transient conditions, Elias et al. [25] specifically suggested using a nonlinear component map modelling strategy that is incorporated with an engine model and supported by the concept of performance adaptability. To simulate actual operations, a controller model that keeps the target temperature (the temperature at the inlet or exhaust of the turbine) and the constant rotational speed constant was utilized. The time-dependent changes in engine parameters such as power, rotational speed, fuel, temperature, and guide vane angles were compared with field data. The operation data and the simulation results were fairly matched. Jeong et al. [26] created a program to simulate the transient behaviour of heavy-duty gas turbines. In this work, an actual load-following operation was simulated, and the effects of ramp rate and load change magnitude were investigated. Li [27] discussed a diagnostic method for a two-shaft gas turbine using transient data. Analysing and comparing the features of the cumulative deviation with traditionally defined performance deviation under steady state conditions has been performed. During defect diagnosis, typical transiently measurable gas turbine engine parameters are used, and a typical slam acceleration process from idle to maximum power is selected for the analysis. The model engine implanted with three typical single-component problems demonstrates the effectiveness of the established diagnostic approach. For gas turbine aero engines, Zhiyuan et al. [28] proposed a self-improving active transient protection control approach based on model-based strategies with the goal of proactive handling of the surge margin limit and turbine entry temperature limit throughout the life cycle. Liuxi et al. [29] examined a transient flow and thermal-fluid-solid coupling to analyse the transient flow and the temperature field of the blade under start-up, shutdown, and varied load conditions.

To assure real-time monitoring of gas turbine performance, Jingchao et al. [30] presented a novel gas-path diagnosis method based on model-data hybrid drive, a mathematical process that involves forward solving. The extracted two-dimensional entropy values shift slightly as the operating conditions change, but the diagnostic results still perform well in terms of interclass separation and intraclass aggregation. Bing et al. [31] suggest a novel approach based on the particle swarm optimization (PSO) algorithm and variable replacement method (VRM) to enhance the control performance in the transient state of a gas turbine engine. The results show that the optimised controller can monitor the acceleration command quickly and precisely while accomplishing the requirement of a minimum fuel ratio unit in deceleration. To determine the relationship between the operating conditions and the cycle parameters, a comprehensive nonlinear thermodynamic model for a single-shaft gas turbine engine is developed by Houman et al. [32]. The results clearly show the trends of both the short-term recoverable deterioration due to fouling effects in the compressor and the long-term non-recoverable deterioration caused by structural degradation. The method is particularly helpful for prognostic applications since it eliminates the need for extra sensors. The effect of ambient parameters on the performance of a combined-cycle gas turbine power plant was modelled and simulated by Marcin and Henryk [33]. With the aid of correction curves, the effect of the variation of ambient temperature on gas turbine electrical power, flue gas stream, and outlet temperature is modelled. A heat recovery steam generator’s mathematical model is based on equations for mass and energy as well as extra equations that define the heat transfer process. On the basis of measurement data, unknown parameters that appear in empirical equations have been estimated. A balance model and a theoretical-empirical model of the steam expansion line in the turbine are included in the steam turbine model. Principles that should be followed in the creation of the compressor cleaning cycle from the viewpoints of safety and economy are proposed, and a matching optimization model is developed by Yunshan et al. [34]. Finally, mathematical calculations were used to examine the effects of combined cycle load and compressor performance degradation rate on the optimal washing cycle. The findings indicate that the aforementioned variables have a significant impact on the optimal washing cycle, and compressor performance degradation rate has the biggest impact.

Sangjo [35] suggested a data-driven modelling approach, considering the time delay included in the measured temperature from a sensor, to create a high prediction accuracy during dynamic operation. Theoklis et al. [36] worked on the gas turbine secondary air system’s transient modelling and simulation. The secondary air system of a two-spool turbofan engine is studied and simulated in transient mode using a flow network modelling approach. Under two predefined scenarios for step and scheduled boundary condition variations, the coupling effect between volume packing and swirl is taken into account in the simulation. In a highly dynamic environment, Tihomir et al. [37] created a transient engine status monitoring model for tiny gas turbines. Adel [33] developed an industrial gas turbine fault diagnosis model using a multi-feedforward artificial neural network and a thermodynamic approach with the aim of improving energy efficiency. Djordje et al. [19] developed a real-time model for predicting gas turbine transient performance. Basic transient phenomena, including volume packing and heat transfer between the working fluid and the structural parts, are included in the method. By solving ordinary differential equations with suitable beginning and boundary conditions, the dynamics of components are quantified. From related performance maps that were previously developed using complex aerodynamic and through-flow codes, compressor and turbine operating points are obtained.

After a comprehensive review, compared with the steady state, the literature on transient analysis is limited. The available transient studies focused on single- and double-shaft configurations. Moreover, the practical system response of transient loads ramping down with physical faults for three shaft-gas turbine configurations has not been investigated. From the diagnostic perspective, it is also important to demonstrate how magnified fault-induced measurement deviations are in transient conditions compared with steady-state conditions. This study aims to address the aforementioned gaps. Before initiating the transient simulation, the performance model has been developed using commercial software [38,39], taking into account variable inlet guide vanes and secondary air system effects. The design point and off-design performance model have been validated with the engine manufacturer’s data. The effects of physical faults such as fouling and erosion on gas turbine performance and gas path measurement deviation have been investigated. The impact of physical faults was studied in both steady and transient states. The comparison has been conducted, and the study proves that the effect of fouling and erosion on steady-state gas turbine performance differs a little from the transient mode of gas turbine performance. It is observed that the deviation is somehow higher in the transient mode than in the steady-state mode of simulation.

## 2. Design Point and Off-Design Performance Model

**Figure 1:**
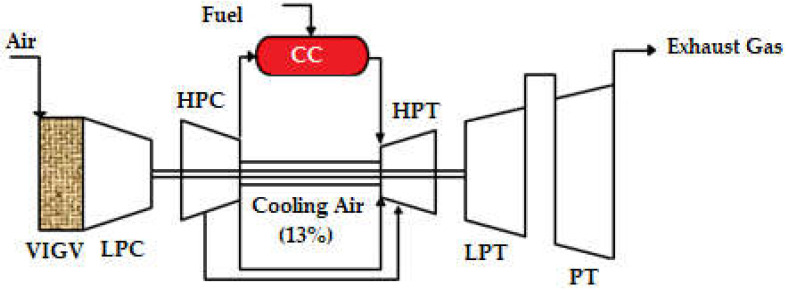
Three-shaft gas turbine configuration.

A three-shaft gas turbine engine, shown in Figure 1, was used as the study’s case engine. It consists of six primary components, which include a low-pressure compressor and turbine, a high-pressure compressor and turbine, a power turbine, and a combustion chamber. It has a single-shaft power turbine and a two-shaft gas generator. The gas generator includes a low- and high-pressure compressor and turbine. The low-pressure turbine and high-pressure turbine are driven by the low-pressure compressor and the high-pressure compressor, respectively. The power turbine has two stages and is a free-axial turbine. The middle and last stages of the high-pressure compressors are used to extract the cooling air.

The gas turbine performance model has been developed by numerous researchers using various techniques. The most accurate way to evaluate gas turbine performance is to calculate enthalpies and entropies at several gas path points. This approach is very powerful as it considers the deviation of specific heat due to the inlet conditions such as ambient temperature, ambient pressure, and humidity [40]. Design point and off-design model development are the key tasks of developing the gas turbine performance model [41]. The design point model computes all parameters at each component station while simulating the engine running at design load. A design point is considered one operating point in off-design performance simulation. Most of the engine data, such as thermal efficiency, power output, exhaust temperature, spool speeds, heat rate, inlet mass flowrate, pressure ratio, compressor, and turbine stages, were gathered from the datasheets of the engine, which were provided by the engine manufacturers. The remaining input parameters were acquired from academic journal articles, and some engineering judgements and approximated values were applied during model optimization. The data to be calculated in the design point simulation included thermodynamic parameters such as P and T at different gas path points, fuel flow rate, air flow rate, component isentropic efficiencies, gas generator speed, net power output, heat rate, specific fuel consumption, and spool speeds. Some of the parameters obtained in the manufacturer’s technical report are depicted in Table 1, and the remaining parameters were calculated using constraints, optimization, and figure of merit parameters depicted in Table 2, Table 3 and Table 4, respectively.

**Figure 2:**
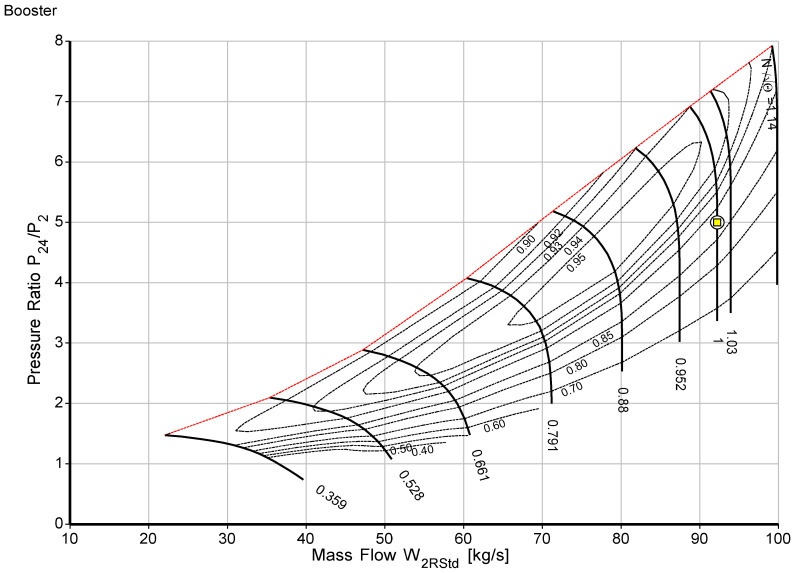
Booster/low-pressure compressor scaled map.

**Figure 3:**
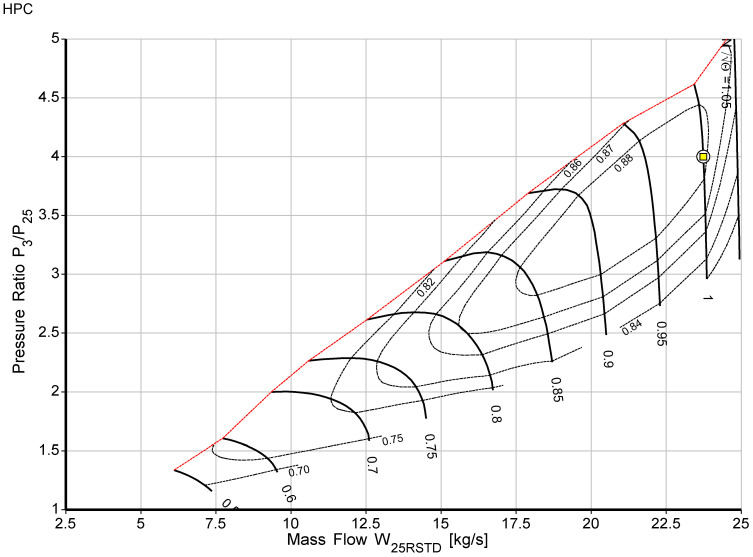
High-pressure compressor scaled map.

The off-design model was built after the cycle design point calculations were successfully developed. The ability of an engine to operate in all operating circumstances, including the design point, is known as “off-design.” The two main factors are engine load and ambient state change. For example, the environment may change significantly from winter to summer, which has a significant impact on engine performance. Adapting the target engine’s design point to the standard compressor and turbine map using the scaling approach is the first stage in the off-design simulations. Each of the five component maps has a design point that was scaled systematically. The Newton-Raphson iterative technique is used in component matching; it is the second stage of off-design, to ensure compatibility with mass flow and work [42,43]. In this study, using commercial software [38,39], the performance model for this study was developed. The software operates by determining the enthalpy and entropy values at each gas path point, which is the most accurate and promising technique. Multiple sources were used to compile the input data for the design point and off-design performance models as well as for model validation. The bleed air and variable inlet guide vane were considered during development. During the optimization process, the target engine component maps were scaled and adapted to the engine design point. The scaled maps for low-pressure and high-pressure compressors are shown in Figure 2 and Figure 3, respectively. The red dashed line in both the high pressure and low-pressure compressors is the surge line, and the yellow square on the map is the design and operating point. Table 5 and Table 6 show components’ maps of coordinate beta and surge margins, and Table 7 shows the component polytropic efficiencies.

**Table 1:** RB211-24G engine design point model input data (sources: datasheet and [44]).

Parameter	Unit	Value
Pressure ratio	-	20:01
Power output	MW	26.025
Thermal efficiency	%	35.8
Heat rate	kJ/kWh	10043
Exhaust mass flowrate	kg/s	92.2
Exhaust temperature	°C	488
HPC rotational speed	RPM	9445
LPC rotational speed	RPM	6643
FPT rotational speed	RPM	4950
HPC stages	-	6
LPC stages	-	7
LPT stages	-	1
HPT stages	-	1
FPT stages	-	2

**Table 2:** Parameters used as constraints.

Parameters	Minimum Value	Optimized Values	Maximum Value
Heat rate (kJ/kWh)	10,026	10,043.3	10,043.5
Thermal efficiency (%)	34	35.85	39
Exhaust temperature (K)	740	751.043	780

**Table 3:** Parameters to be optimized.

Variables	Min Value	Optimized Value	Max Value
HPT Rotor 1 Cooling Air	0.06	0.075	0.085
HPT NGV 1 Cooling Air	0.04	0.055	0.065
Exhaust Pressure Ratio	1	1.144	1.2
IPC Isentropic Efficiency	0.87	0.8789	0.91
HPC Isentropic Efficiency	0.88	0.8822	0.9
HPT Isentropic Efficiency	0.8836	0.8945	0.9
PT Isentropic Efficiency	0.8999	0.9027	0.91
LPT Isentropic Efficiency	0.91	0.9247	0.93

**Table 4:** Objective function.

Parameter	Value
Power output (kW)	26,025

**Table 5:** Map coordinate beta.

No.	Components	Map Coordinate Beta
1	LPC	0.4
2	HPC	0.5
3	HPT	0.5
4	LPT	0.5
5	PT	0.7

**Table 6:** Compressor surge margin.

No.	Compressors	Surge Margin in [%]
1	LPC	56.5
2	HPC	24.1

**Table 7:** Component polytropic efficiency.

Component	Optimized Polytropic Efficiency
Booster/low-pressure compressor (LPC)	0.90
High-pressure compressor (HPC)	0.90
High-pressure turbine (HPT)	0.88
Low-pressure turbine (LPT)	0.89
Power Turbine (PT)	0.91

The design-point performance model was developed after several iterations. The developed model was validated with the engine datasheet. The secondary air system and some engineering judgements based on published papers have been used. First, all known parameters were filled into the design point input window, and the simulation was initiated to see the model on the first attempt. Since getting an accurate model on the first simulation attempt is unlikely, the optimization task was conducted until the model output results matched the engine datasheet.

Even though surge likely occurs in the compressor and choking likely occurs in the turbine, Table 5 and Table 6 show the scaled component map information that describes how the engine performance model has no issue with surging or choking. All the component map beta values shown in Table 5 are where the design point is placed. Table 6 shows the maximum surge percentage of the low-pressure compressor and the high-pressure compressor.

Finally, the design parameters from the gas turbine product datasheet were compared with the output of the design point model, as shown in Table 8. The design point values generated by the model developed using commercial software were determined to be acceptable due to their agreement with the real design values and very low discrepancy.

**Figure 4:**
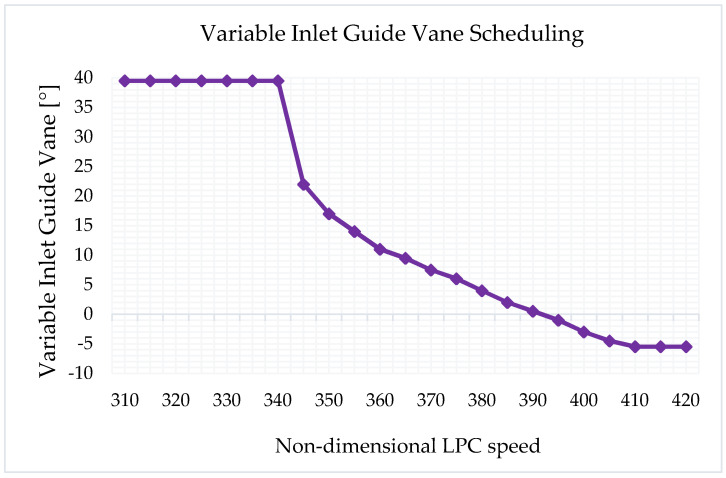
RB211−24G VIGV’s schedule (N_1_/√T_0_).

The off-design model was created and validated using manufacturer data after the component maps were scaled and the cycle design point was correlated. The variable inlet guide vane schedule is shown in Figure 4. Likewise, the design point model was validated with the engine datasheet data, and the off-design model was also validated with engine manufacturer curves such as efficiency versus ambient temperature and power output versus ambient temperature.

**Figure 5:**
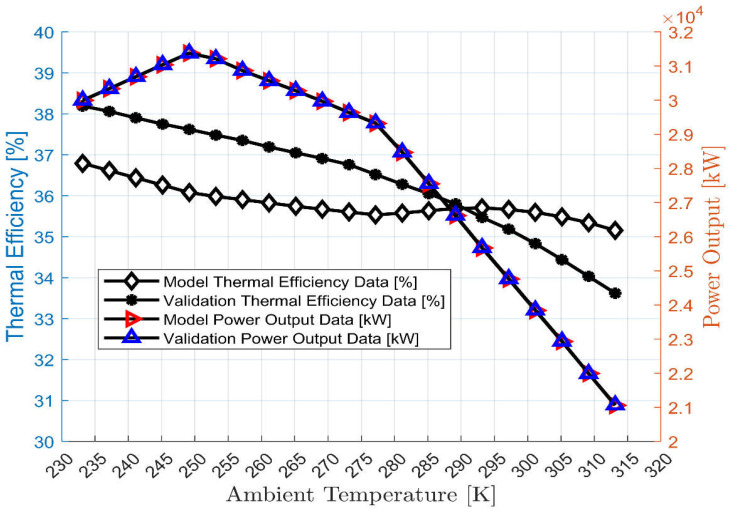
Validation of the off-design model.

**Figure 6:**
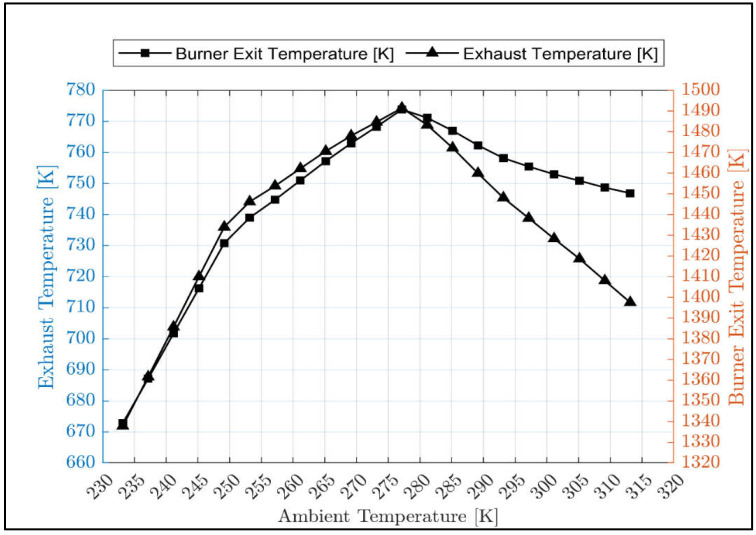
Exhaust temperature and burner exit temperature versus ambient temperature.

The off-design model was used to generate data after incorporating the power output versus ambient temperature and VIGV scheduling into the software. The generated data were then compared with the validation data. The validation data and the model that produces the efficiency versus ambient temperature and power output versus ambient temperature were closely matched. At each operational point, the power output versus ambient temperature error from the validation data was 0.02%, while a 4.05% error was found for the efficiency versus ambient temperature output data, as shown in Figure 5 and Figure 6, respectively.

Figure 5 showed that the result of the off-design model closely matched the validation data. This shows that the model is accurate and acceptable for predicting the performance of the gas turbine under various operating conditions. Using the validated off-design model, the burner exit temperature and the gas turbine exhaust temperature have been simulated and shown in Figure 6. The trend of exhaust temperature versus ambient temperature is the same as the actual exhaust temperature versus ambient temperature trend of another three-shaft engine [45].

## 3. Transient Simulation

**Figure 7:**
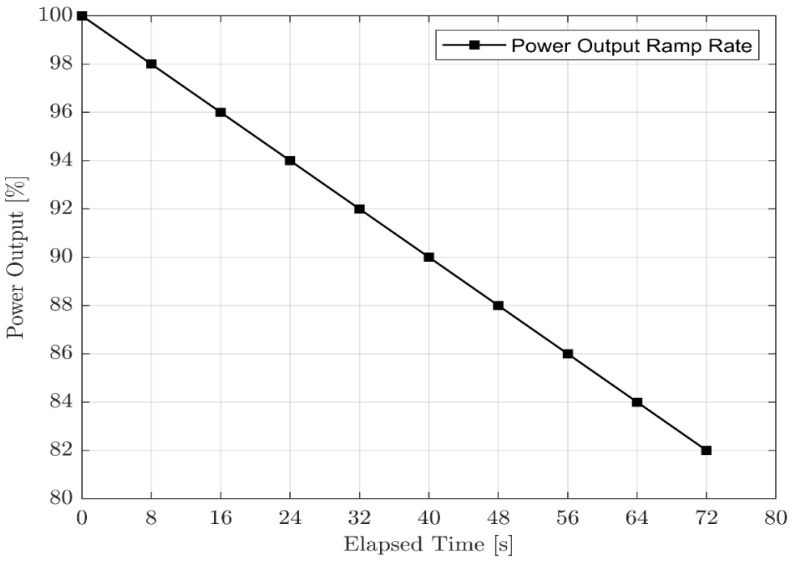
Power ramp down schedule in transient simulation.

In transient simulation, only a few input variables are required if the scope is performance. The polar moment of inertia is the fundamental difference between transient simulation and steady-state. The rest of the additional details on the transient input page describe a conventional proportional–integral differential control system. The actual transient simulation begins with steady-state operation, which has been calculated as a single cycle. The transient behaviour of a gas turbine engine can be simulated based on fuel flow rate versus time control, power output versus time control, and spool speed versus time control. In this study, power output versus time control was used, and a practical power ramp-down rate that was found in the research paper [2] was used. Since the software used allows for a transient operation with a maximum time span of 72 s, the power output was stepped down to 82% from 100%. It is the practical power output versus time schedule in 72 s. The power step-down rate is 2% [16]. The power output versus elapsed time control that was used during the transient simulation is shown in Figure 7.

### PID Control

In practice, the transient operating point of the gas turbine is achieved by modulating the fuel flow rate. In turn, a control system compares the generated power output with the demanded power output based on the signals provided by the sensors. Any discrepancy between the demanded power and generated power leads to either acceleration or deceleration. The commercial software that we used in this study uses a very simple controller named Proportional Integral Derivative (PID). The PID controller increases or decreases the fuel flow rate to maintain the demanded power. The PID uses the spool speed as input to navigate the fuel flow rate. Generally, a Proportional Integral Derivative (PID) controller works by controlling an output to bring a process value to a desired set point. The PID controller is continuously monitoring the error value and, using this value, calculating the proportional, integral, and derivative values. The controller then adds these three values together to create the output. In a PID controller, there is a term called “gain”. It is the term used for the multiplication factor. By adjusting the gain settings or multiplication factor of the proportional, integral, and derivative, the user can control how much effect the PID controller has on the output and how the controller will react to different changes in the process value. Each of the terms, proportional (P), integral (I) and derivative (D) will be calculated to get the output. The proportional term is calculated by multiplying the P-gain by the error. The purpose of the proportional is to have a large immediate reaction on the output to bring the process value close to the set point. Due to the limitation of the P-controller, where there is always an offset between the process variable and setpoint, an integral term is needed, which provides the necessary action to eliminate the steady-state error. It integrates the error over a period of time until the error value reaches zero. It holds the value for the final control device at which error becomes zero. The last one is the derivative term that creates an output signal proportional to the rate of change of the error signal. The derivative term looks ahead to see what the error will be in the future and contributes to the controller’s output accordingly. That brings us to a term called “controller tuning”.

The commercial software that we used in this study uses Equations (1)–(3) to express Proportional Integral Derivative (PID). The fuel acceleration or deceleration is caused by any difference between the generated and required power. That means the fuel flow will be either increasing or decreasing based on the discrepancies between the actual and desired power output values.

(1) $$\Delta m_{fP}=K_{P}\left(N_{demand}-N\right)$$

(2) $$\Delta m_{fI}=K_{I}\int \left(N_{demand}-N\right).dt$$

(3) $$\Delta m_{fD}=K_{D}\frac{d\left(N_{demand}-N\right)}{dt}$$

where $\Delta m_{f}p,\;\Delta m_{f}I$ and $\Delta m_{f}D$ is the fuel change due to the difference between demand power and generated power at proportional, integral, and derivative control, $N_{demand}$ is the demanded speed, N is the actual speed, and $K_{P},K_{I}\;\mathrm{and}\;K_{D}$ are gains called the proportional control constant, the integral control constant, and the differential control constant, respectively. Such optimal gains used in the PID controller help minimize the difference between the generated and demanded power. Table 9 shows the transient control data that were used during the simulation. Some of the values in the table were estimated and iterated until the generated and demanded power matched. The values of the shaft moment of inertia for a three-shaft gas turbine were collected from the published paper [18].

## 4. Fouling and Erosion Simulation

The performance of the gas turbine components, especially the compressor and turbine, is essential to the engine’s overall performance because of its vulnerability to various internal and external deterioration factors. A lack of lubricating oil may be the primary factor in mechanical problems, which also include misalignment, imbalance, cracks, and bearing failures. Performance-related problems such as dust and fouling in compressors, corrosion, erosion, inappropriate combustion, increased clearance around blade tips, domestic object damage (DOD), and thermal distortion are the second cause of gas turbine deterioration. There are two types of gas turbine performance degradation: temporary (recoverable by washing) and permanent (non-recoverable by washing). Temporary degradation can be recovered during engine overhaul and operation; however, permanent degradation requires replacement. Temporary degradation is caused by fouling, erosion, corrosion, and blade tip clearance. The most common causes of gas turbine performance drops are fouling and erosion [46]. Fouling, which typically accounts for more than 70% of the overall degradation of engine performance over the period of operation, is one of the most common causes of component deterioration [47,48,49]. Thus, in this paper, fouling and erosion were simulated in the transient operation. The relationship between physical faults and the health parameter presented in Table 10 was used. 

Following the validation of the design point and off-design gas turbine performance models in this study, the next task was to implant physical faults such as fouling and erosion to investigate their effect on the gas turbine’s performance at steady state and transient state. While introducing physical faults into the model, the relationship between physical faults and performance parameter deviation shown in Table 10, which was experimentally investigated and found in [50,51], was used. The up arrow shows that the health parameters are increasing whereas the down arrow shows decreasing. As depicted in Table 10, during the simulation of compressor fouling, the ratio of flow capacity to isentropic efficiency is 3:1, whereas the ratio is 2:1 in the simulation of compressor erosion, turbine fouling, and erosion. Flow capacity increases when turbine erosion occurs. Both fouling and erosion are simulated at 100% fault severity, which means that during compressor fouling, flow capacity and isentropic efficiency were decreased by −7.5% and −2.5%, respectively, whereas they were decreased by −4% and 2% during compressor erosion and turbine fouling. However, during turbine erosion, the flow capacity increased by 4% and the isentropic efficiency decreased by 2%.

## 5. Results and Discussion

### 5.1. Transient Operation: The Effect of Fouling and Erosion on the Component Isentropic Efficiency

**Figure 8:**
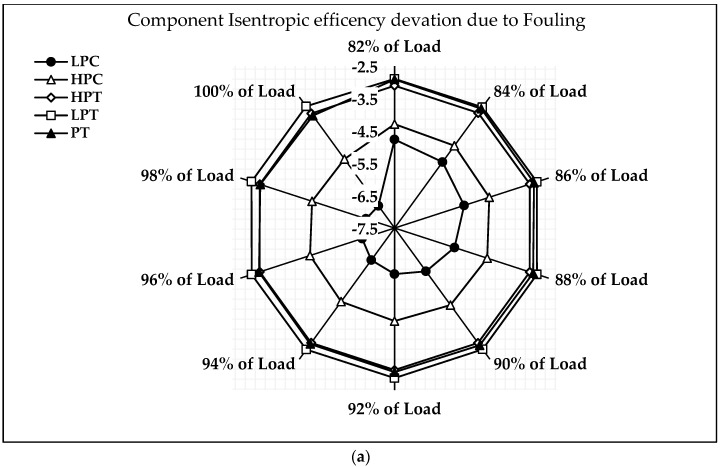

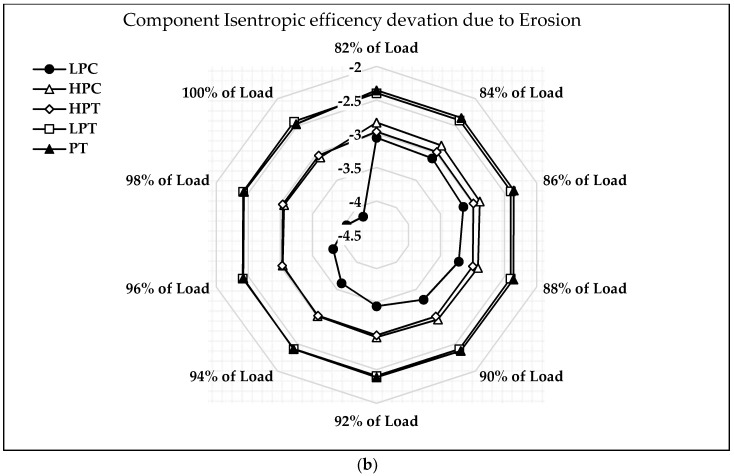
Deviation of component isentropic efficiency: (**a**) Fouling, (**b**) Erosion.

Figure 8a shows the deviation of component isentropic efficiency when fouling occurs at 100% fault severity with load variation during transient operation. The LPC isentropic deviation due to LPC fouling is −6.6 percent at full load and −4.6% at 82% load. The deviation of HPC isentropic efficiency due to HPC fouling at 100% load is −4.86%, whereas it is −4.3% at 82% load. The deviation of HPT isentropic efficiency due to HPT fouling at 100% load is −3.12%, whereas it is −3.1% at 82% load. The deviation of LPT isentropic efficiency due to LPT fouling at 100% load is −2.85%, whereas it is −2.89% at 82% load. The deviation of PT isentropic efficiency due to PT fouling at 100% load is −3.21%, whereas it is −2.91% at 82% load. The results show that the isentropic efficiency deviation of a LPC due to fouling is higher than other component deviations. It is also observed that the deviation of LPC, HPC, HPT, and PT isentropic efficiency due to the LPC physical faults decreases as the load decreases, whereas it increases in the PT. It is clearly observed that the occurrence of a physical fault has a greater effect on the upstream components than the downstream components. 

Figure 8b shows the deviation of component isentropic efficiency when erosion occurs at 100% fault severity with load variation during transient operation. The isentropic efficiency deviation of LPC due to erosion at full load is −4.16% and −3.05% at 82% load. The deviation of HPC isentropic efficiency due to erosion at 100% load is −3.07%, whereas it is −2.83% at 82% load. The deviation of HPT isentropic efficiency due to erosion at 100% load is −3.04%, whereas it is −2.96% at 82% load. The deviation of LPT isentropic efficiency due to erosion at 100% load is −2.42%, whereas it is −2.39% at 82% load. The deviation of PT isentropic efficiency due to erosion at 100% load is −2.46%, whereas it is −2.35% at 82% load. It is again observed that the isentropic efficiency deviation of a LPC due to erosion is higher than other component deviations. Following the simulation of the effect of physical faults on the component’s isentropic efficiency at the transient mode of operation, the same pattern of physical fault was injected into the clean engine model, and its effect on the engine was investigated. The ten best gas path diagnosis parameters for both the transient and steady state modes of operation were also investigated. These are the ten best three-shaft engine diagnosis parameters suggested by Mohd et al. [44]. The suggested gas path measurement parameters are PT4, T24, P3, T3, P43, P47, T5, FF, N1, and N2.

### 5.2. Transient Operation: The Effect of Fouling and Erosion on the Gas Path Measurement Parameters

**Figure 9:**
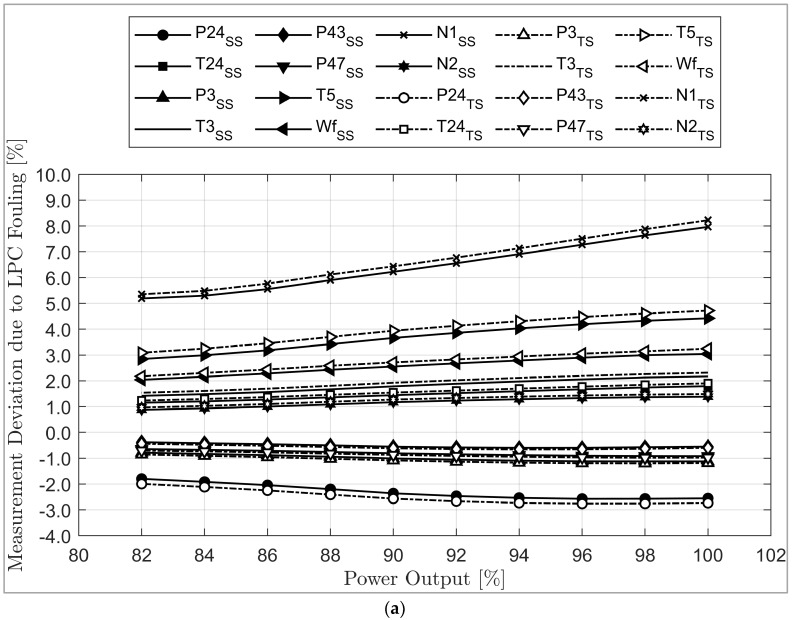

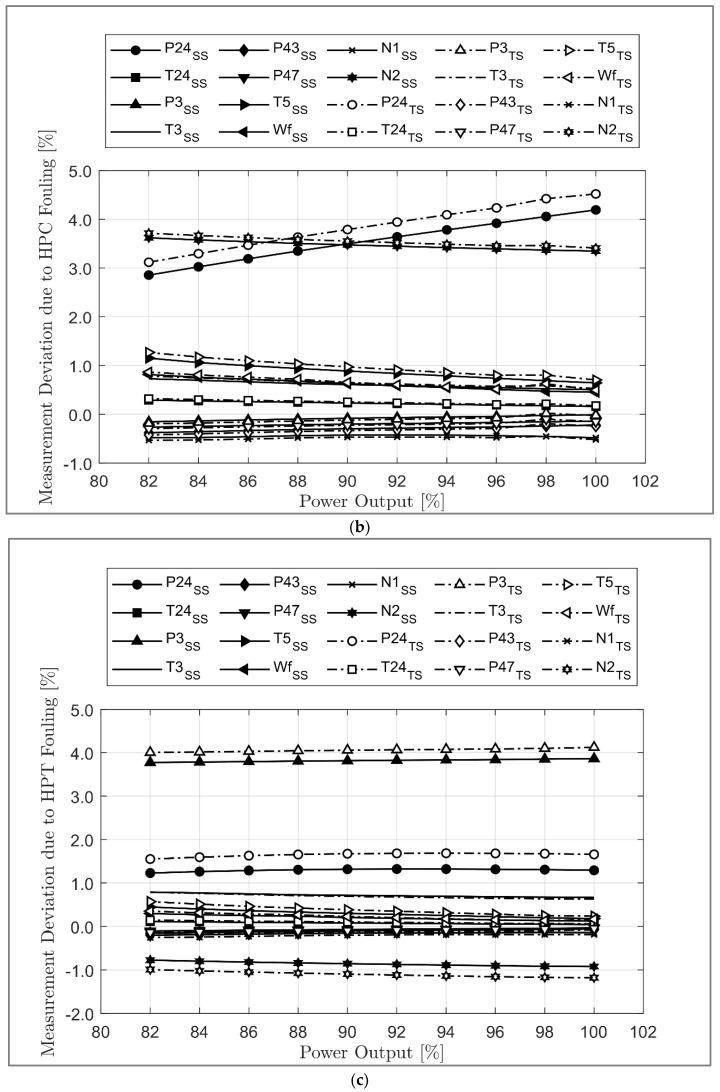

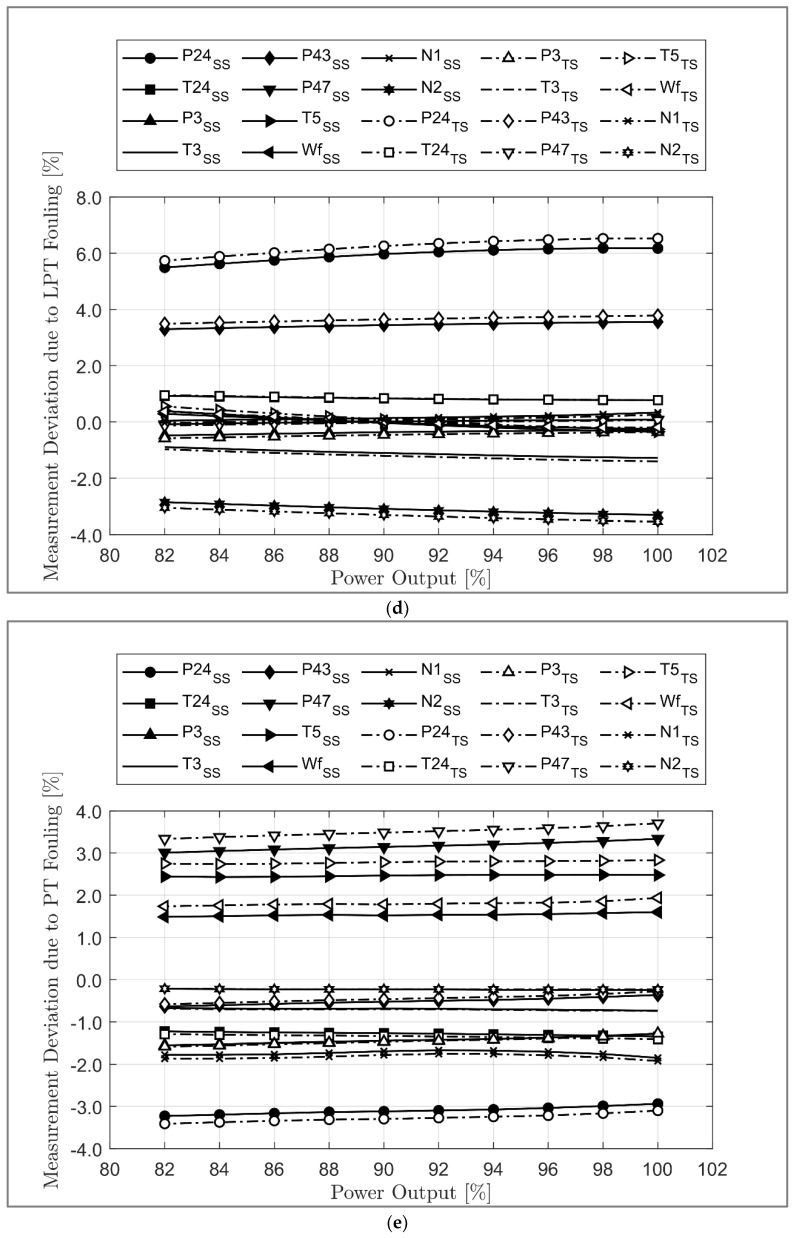

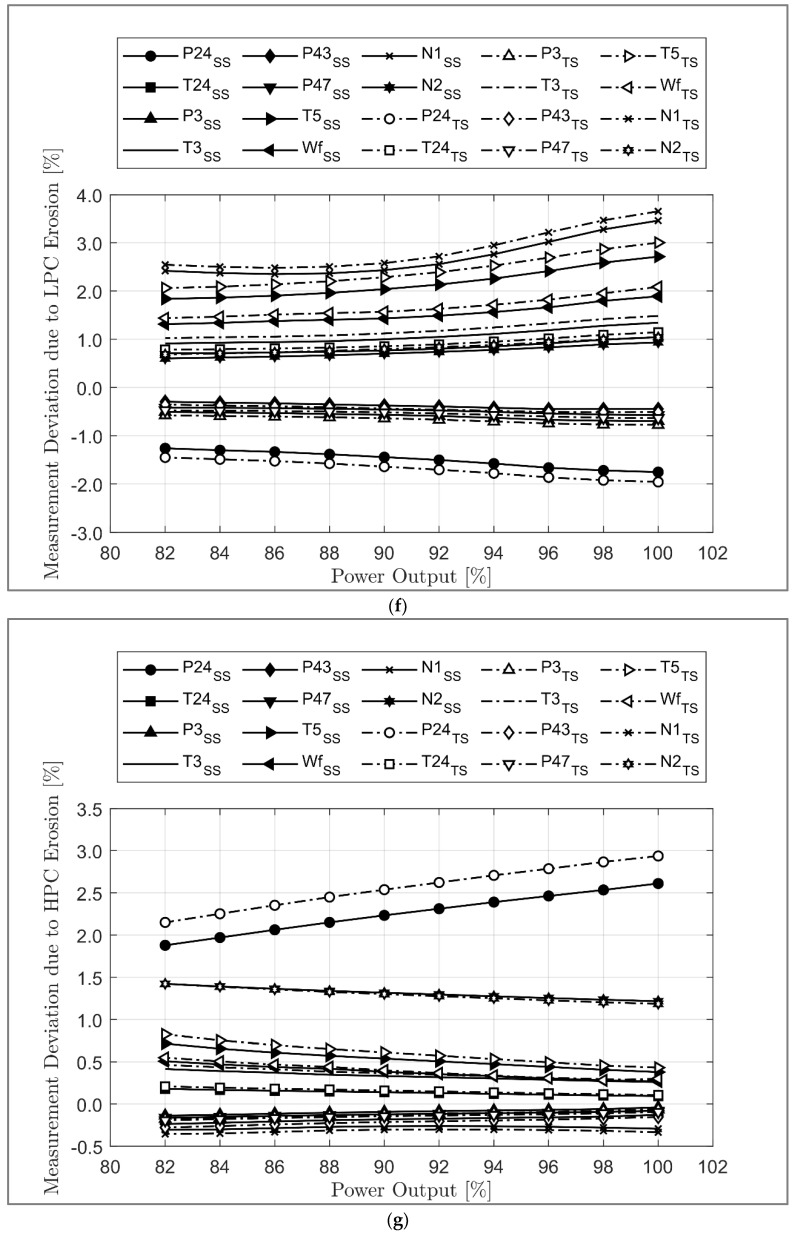

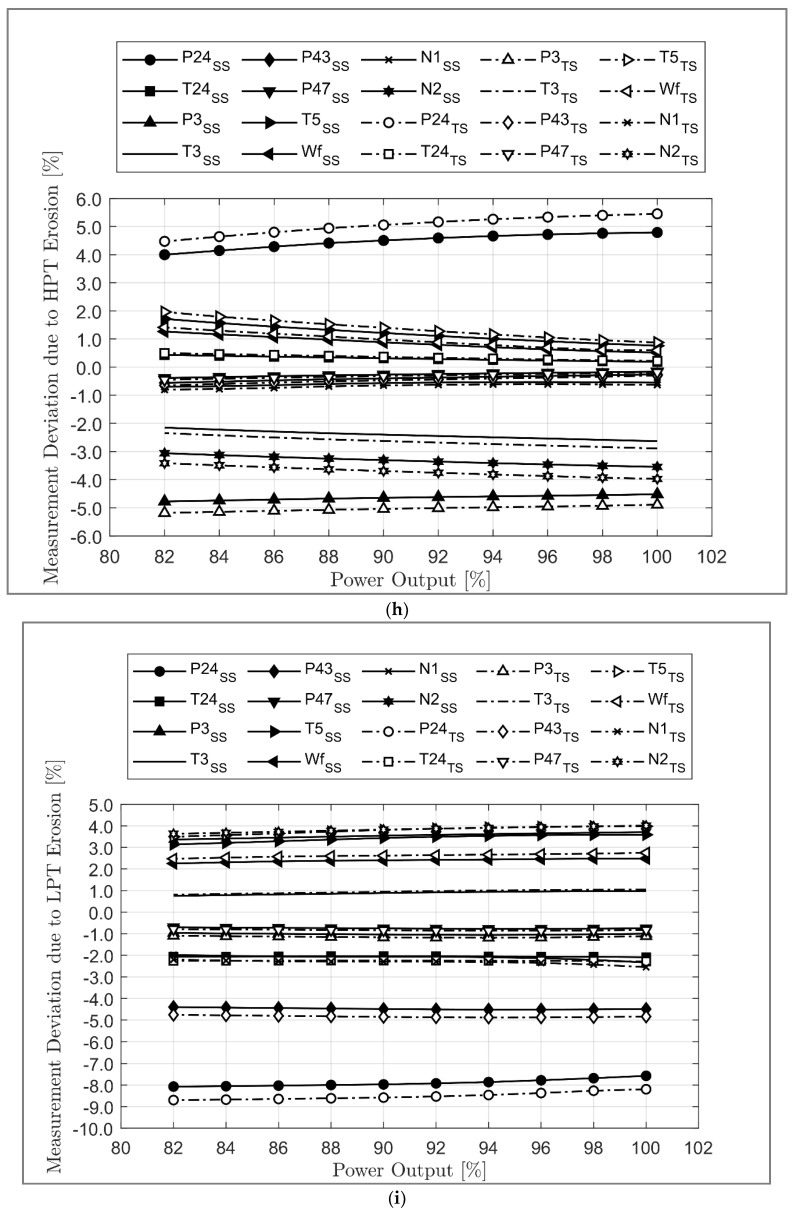

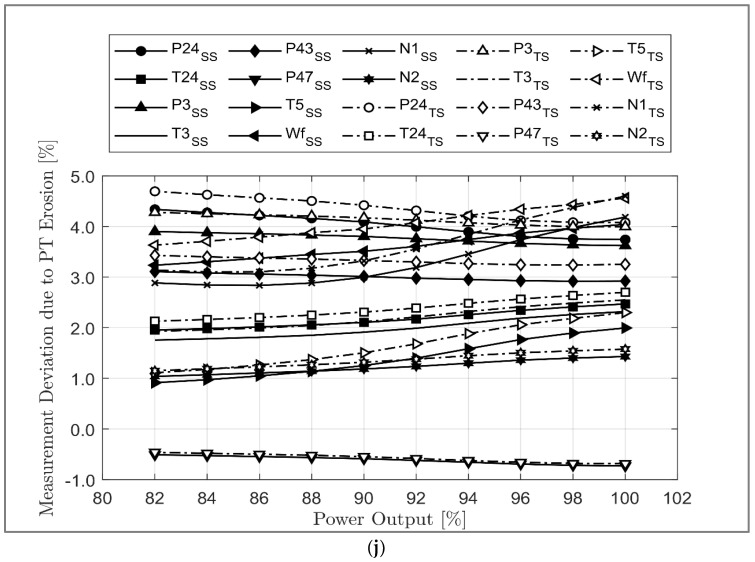
Deviation of component measurement parameters: (**a**) LPC fouling, (**b**) HPC fouling, (**c**) HPT fouling, (**d**) LPT fouling, (**e**) PT fouling, (**f**) LPC erosion, (**g**) HPC erosion, (**h**) HPT erosion, (**i**) LPT erosion, (**j**) PT erosion.

Figure 9a–e show the deviation of gas path measurement when fouling occurs at 100% fault severity with load variation during transient operation. As shown in Figure 9a, due to LPC fouling, N1, T5, WF, P24, and T3 significantly deviated from the clean condition. The deviation decreases as the load decreases. The difference between the deviation that occurred in steady state and transient modes of simulation is too small in all gas path parameters. Figure 9b shows the effect of HPC fouling. The most deviated parameters are N2 and P24. Except in P24, all gas path measurement parameter deviations increase as the load decreases, and the difference between the deviations occurring in steady state and transient modes of simulation seems significant in P24, about 0.33% at 100% load and 0.26% at 82% load. In Figure 9c, the effect of HPT fouling is shown. Due to HPT fouling, the two most deviated parameters are P24 and P3. Except for N2, the deviation increases as the load decreases. The difference between the deviation that occurred in steady state and transient modes of simulation seems significant in P24, P3, and N2, at about 0.37%, 0.26%, and 0.256% at 100% load, and 0.32%, 0.23%, and −0.21% at 82% load, respectively. Figure 9d shows that, due to LPT fouling, the most deviated parameters are P24, P43, T3, and N2. Except for T24, P3, T5, and WF, the deviation decreases as the load decreases. The difference between the deviation that occurred in steady state and transient modes of simulation seems significant in P24 and N2, about 0.35% and −0.24% at 100% load and 0.24% and −0.2% at 82% load, respectively. Figure 9e shows that, due to PT fouling, the most deviated parameters are P24, P3, and T5. Except for P24, T24, P3, and P43, the deviation decreases as the load decreases. The difference between the deviation that occurred in steady state and transient modes of simulation seems significant in P47, about 0.367% at 100% load and 0.33% at 82% load, respectively.

The deviation of gas path measurement when erosion occurs at 100% fault severity with load variation at transient operation is shown in Figure 9f–j. Due to LPC fouling, N1, T5, WF, P24, and T3 significantly deviated from the clean condition. It is shown in Figure 9f. The deviation decreases as the load decreases. The difference between the deviation that occurred in steady state and transient modes of simulation seems significant in P24, at about −0.2% at 100% load and −0.19% at 82% load. Figure 9g shows the HPC erosion effect. The result shows that the most deviated parameters are N2 and P24. Except in P24, all gas path measurement parameter deviations increase as the load decreases, and the difference between the deviations occurring in steady state and transient modes of simulation seems significant in P24, at about 0.32% at 100% load and 0.27% at 82% load. Figure 9h shows the HPT erosion effect. The most deviated parameters are P24 and P3. Except for P24, T3, and N2, the deviation increases as the load decreases. The difference between the deviation that occurred in steady state and transient modes of simulation seems significant in P24 and N2, about 0.67% and −0.43% at 100% load and 0.47% and −0.35% at 82% load, respectively. Figure 9i shows the effect of LPT erosion. The most deviated parameters are P24, T24, P43, T5, WF, N, and N2. Except for P24, the deviation decreases as the load decreases. The difference between the deviation that occurred in steady state and transient modes of simulation seems significant in P24, P43, T5, and N2 parameters. Figure 9j shows the PT erosion effect. In this case, the most deviated parameters are P24, P3, P43, WF, and N1. Except for P24, P3, and P43, the deviation decreases as the load decreases. The difference between the deviation that occurred in steady state and transient modes of simulation seems significant in P24, P3, and P43 parameters.

## 6. Conclusions

This study clearly shows that physical faults such as fouling and erosion can be detected much earlier in a transient simulation than in a steady state. It is also observed that the effect of fouling and erosion on steady-state gas turbine performance differs a little from the transient mode of gas turbine performance. It is observed that the deviation is somehow higher in the transient mode than in the steady-state mode of simulation. In most cases, as the load increases, the deviation of the performance parameter and measurement parameter increases. However, because of component matching discrepancies, in some cases, the deviation of the gas path and performance parameters are not linear with the load ramp rate. The simulation result shows that fouling has a significant effect on the upstream components more than the downstream components, and the health status of the upstream components is very critical to the entire performance of the gas turbine. The maximum isentropic efficiency deviation due to fouling and erosion occurs in the low-pressure compressor more than in other components. The fuel flow rate is raised when fouling and erosion occur at each component and at each power ramp rate. The reason is that when physical faults are injected by manipulating flow capacity and isentropic efficiency at power output versus ambient temperature scheduling, the values of the output flow capacity and isentropic efficiency will be reduced significantly more than the amount reduced to simulate the physical faults. This is because more fuel must be delivered to maintain the power output. The increase in heat at constant power causes decreases in component efficiency and overall gas turbine efficiency as well. Generally, in this paper, three-shaft gas turbine transient performance gained the focus. The deviation of component isentropic efficiency and gas path measurement parameters under transient mode are discussed following the plots. The results will be very helpful while developing a fault detection, isolation, and identification model. The authors suggest that future research could look into the start-up performance of three-shaft gas turbines, which is beyond the scope of this study. Furthermore, the combined effect of physical faults in a transient state remains an unexplored problem for researchers to investigate.

## Figures and Tables

**Table 8:** The design point model output.

Parameter	Units	Datasheet and [39]	Model	% Error
Power Output	kW	26,025	26,025.1	0
Thermal Efficiency	%	35.8	35.84	0.1
Pressure ratio	-	20:1	20:1	0
Fuel Flowrate	kg/s	-	1.696	-
Lower Heating Value	MJ/kg	42.8	42.8	0
Exhaust Temperature	°C	488	478	1.3
Heat Rate	kJ/(kW·h)	10,043	10,043.3	0

**Table 9:** Transient input data.

Transient Parameters	Units	Value
LP Spool Polar Moment of Inertia	kg·m^2^	2.1
HP Spool Polar Moment of Inertia	kg·m^2^	0.014
PT Spool Polar Moment of Inertia	kg·m^2^	2.4
Proportional Control Constant, $K_{P}$		1
Integral Control Constant, $K_{I}$		0
Differential Control Constant, $K_{D}$		0.5
100% Overboard Bleed	kg/s	0.3
Minimum Fuel–Air Ratio		0.01
Maximum Fuel–Air Ratio		0.04
Deceleration Limit		0.1
Acceleration Limit		0.1
Temperature Sensor Time Constant		0.5
Burner Time Constant		0.1
Maximum Limiter Gain Modifier		1

**Table 10:** Health parameters and the physical fault are related [50,51].

Physical Fault	Flow Capacity Change (A)	Isentropic Efficiency Change (B)	Ratio A:B	Range
Compressor fouling	Γ_C_ ↓	η _C_ ↓	3:1	(0 to −7.5%) (0 to −2.5%)
Compressor erosion	Γ_C_ ↓	η _C_ ↓	2:1	(0 to −4%) (0 to −2%)
Turbine fouling	Γ_T_ ↓	η _T_ ↓	2:1	(0 to −4%) (0 to −2%)
Turbine erosion	Γ_T ↑_	η _T_↓	2:1	(0 to +4%) (0 to−2%)

## Data Availability

Data available on request due to restriction.

## Nomenclature

HPC	High-pressure compressor
LPC	Low-pressure compressor
LPT	Low-pressure turbine
HPT	High-pressure turbine
PT	Power turbine
T24	Low-pressure compressor exit Temperature
P24	Low-pressure compressor exit Pressure
P3	High-pressure compressor exit pressure
P43	High-pressure turbine exit pressure
T3	High -pressure compressor exit Temperature
P47	Low-pressure turbine exit pressure
FF	Fuel flow
T5	Power turbine exit temperature
N1	Low-pressure speed
GT	Gas Turbine
N2	High-pressure speed
CC	Combustion chamber
VIGV	Variable inlet guide vane
DOD	Domestic object damage
SS	Steady state
TS	Transient state
GPA	Gas path analysis
MB	Model-based
KF	Kalman filter
AI	Artificial intelligence
ANN	Artificial neural network
ES	Expert system
FL	Fuzzy logic
BBN	Bayesian belief network
DL	Deep learning
GA	Genetic algorithm
